# Adipose Tissue Secretion Pattern Influences β-Cell Wellness in the Transition from Obesity to Type 2 Diabetes

**DOI:** 10.3390/ijms23105522

**Published:** 2022-05-15

**Authors:** Giuseppina Biondi, Nicola Marrano, Anna Borrelli, Martina Rella, Giuseppe Palma, Isabella Calderoni, Edoardo Siciliano, Pasquale Lops, Francesco Giorgino, Annalisa Natalicchio

**Affiliations:** Department of Emergency and Organ Transplantation, Section of Internal Medicine, Endocrinology, Andrology and Metabolic Diseases, University of Bari Aldo Moro, 70124 Bari, Italy; giuseppina.biondi@uniba.it (G.B.); nicola.marrano@uniba.it (N.M.); a.borrelli93@gmail.com (A.B.); m.rella22@studenti.uniba.it (M.R.); giuseppepalma1990@yahoo.it (G.P.); i.calderoni1@studenti.uniba.it (I.C.); e18.siciliano@gmail.com (E.S.); lopspasquale@gmail.com (P.L.); francesco.giorgino@uniba.it (F.G.)

**Keywords:** type 2 diabetes, obesity, pancreatic β-cells, adipose tissue, adipokines, cross-talk

## Abstract

The dysregulation of the β-cell functional mass, which is a reduction in the number of β-cells and their ability to secure adequate insulin secretion, represents a key mechanistic factor leading to the onset of type 2 diabetes (T2D). Obesity is recognised as a leading cause of β-cell loss and dysfunction and a risk factor for T2D. The natural history of β-cell failure in obesity-induced T2D can be divided into three steps: (1) β-cell compensatory hyperplasia and insulin hypersecretion, (2) insulin secretory dysfunction, and (3) loss of β-cell mass. Adipose tissue (AT) secretes many hormones/cytokines (adipokines) and fatty acids that can directly influence β-cell function and viability. As this secretory pattern is altered in obese and diabetic patients, it is expected that the cross-talk between AT and pancreatic β-cells could drive the maintenance of the β-cell integrity under physiological conditions and contribute to the reduction in the β-cell functional mass in a dysmetabolic state. In the current review, we summarise the evidence of the ability of the AT secretome to influence each step of β-cell failure, and attempt to draw a timeline of the alterations in the adipokine secretion pattern in the transition from obesity to T2D that reflects the progressive deterioration of the β-cell functional mass.

## 1. Adipose Tissue/β-Cell Cross-Talk: A Bidirectional Communication

Adipose tissue (AT) is a complex organ composed of several cell types, such as adipocytes and the cells of the stromal vascular fraction—including macrophages, and endothelial and blood cells [1]. According to its various characteristics and functions, AT is generally classified into four types: white, pink, beige, and brown adipose tissue. White adipose tissue (WAT) is characterised by high insulin sensitivity and plays a crucial role in energy storage and endocrine communication, while brown adipose tissue (BAT) is involved in the regulation of thermogenesis as it can use energy to produce heat. Between these extremes, the beige (or ‘brite’, brown-in-white) AT, a brown AT raised in WAT, is involved in both thermogenesis and endocrine communication [2]. Finally, the pink adipocyte is a milk-secreting mammary gland alveolar epithelial cell that can arise from transdifferentiation of white adipocytes during pregnancy and lactation, with a great potential for energy storage and a higher metabolic activity compared to white adipocytes [3,4]. Based on its localisation, adipose tissue is also distinguished as visceral adipose tissue (VAT) and subcutaneous adipose tissue (SAT). VAT is metabolically more active than SAT and characterised by greater lipolysis and lower insulin sensitivity. A large increase in VAT is closely related to a higher incidence of metabolic disturbances and mortality [5,6,7].

As mentioned, the traditional role of WAT is to regulate systemic energy homeostasis by storing free fatty acids (FFAs) in triglycerides (TGs) during excess nutrient conditions (adipogenesis), while releasing them during the fasting state (lipolysis) [8]. Insulin is the main regulator of these processes as it inhibits lipolysis, stimulates the absorption of glucose and circulating FFAs in the AT, and promotes the synthesis of TGs [8,9,10]. Overall, insulin is an adipogenic hormone that plays a crucial role in regulating glucose and lipid metabolism.

Given the relevant effects of insulin in the AT, the existence of a communication between AT and pancreatic β-cells has long been known, and, for many years, this relationship has been thought to be unidirectional. To date, AT is considered not only as a depository for excess energy but also as an endocrine organ that produces and releases a large number of hormones/cytokines (referred to as adipokines) and FFAs able to affect the function of many tissues [11,12,13]. These factors are involved in numerous biological processes, including glucose and lipid metabolism, food intake, inflammation, coagulation, and the maintenance of metabolic homeostasis [13]. Significantly, many of these adipokines and FFAs might directly influence numerous aspects of β-cell function and viability, including insulin synthesis and secretion as well as β-cell apoptosis and proliferation [14,15,16]. Evidence suggests that the cross-talk between AT and pancreatic β-cells is bidirectional and could drive the maintenance of the β-cell functional mass under physiological conditions [17,18].

## 2. Dysfunctional Adipose Tissue in Obesity: Alteration of the Adipocyte Secretome

The World Health Organization defines obesity as an excessive fat accumulation that presents a health risk, and it is well documented that it represents one of the major causes of T2D [19]. Indeed, when the dietary fat surfeit exceeds the storage ability of AT, it accumulates in ectopic sites, thus contributing to enlarging visceral deposits and resulting in FFA-induced lipotoxicity. In particular, when ectopic fat accumulates in the pancreas, it could contribute to β-cell dysfunction [20]. Accordingly, recent studies in humans have proved that bariatric surgery can improve β-cell function by decreasing fat accumulation [21], including pancreatic fat [22].

In addition, obesity is associated with an increase in the size and number of adipocytes [23], establishment of a low chronic inflammatory state [24,25], reduction in whole-body insulin sensitivity [26], and increased or decreased levels of adipokines secreted by adipocytes which are reflected in an alteration of their serum levels [27,28,29,30,31]. These features describe a dysfunctional AT and could represent the mechanistic link between obesity and T2D [26,32,33,34].

Compared to the AT of lean individuals, the AT of obese patients produces higher levels of pro-inflammatory adipokines—such as tumour necrosis factor-α (TNF-α), interleukin-6 (IL-6) [35], and monocyte chemoattractant protein-1 (MCP-1) [36]—which promote insulin resistance and strongly contribute to the development of T2D [37,38,39,40]. Accordingly, in obese patients with T2D, IL-6 and TNF-α levels are higher compared to obese, non-diabetic patients [41,42,43]. On the one hand, it is noteworthy that both IL-6 and TNF-α could be secreted by macrophages that infiltrate the AT of obese patients rather than by adipocytes [24,44]. On the other hand, MCP-1 is produced by both adipocytes and stromal vascular cells, and can attract monocytes and leukocytes in response to an inflammatory environment [36].

Furthermore, AT dysfunction is associated with the release of other adipokines with pro-inflammatory, pro-thrombotic, anti-adipogenic, and anti-fibrinolytic effects, such as macrophage inflammatory protein-1α and -1β (MIP-1α and -1β), regulated upon activation, normal T cells expressed and secreted (RANTES or C-C Motif Chemokine Ligand 5, CCL5), growth-related oncogene factor-alpha (GRO-alpha), thrombopoietin (TPO), tissue inhibitor of metalloproteinases-1 (TIMP-1), and plasminogen activator inhibitor-1 (PAI-1) [27,28,45,46,47,48,49,50,51,52]. The levels of these adipokines are higher in obese than in healthy subjects and correlate positively with glycemia and insulinemia, while correlating negatively with insulin sensitivity [28].

Leptin and adiponectin represent the best-known adipokines. Alterations in their levels are well-characterised indicators of AT dysfunction in obesity [29,53]. Leptin plays a key role in regulating glucose homeostasis, increasing energy expenditure and reducing food intake and body weight [54,55]. Animal models lacking leptin or the leptin receptor (*ob/ob* and *db/db* mice, respectively) show weight gain, hyperinsulinemia, insulin resistance, and impaired glucose homeostasis [56]. Conversely, obese subjects have been reported to have higher serum leptin concentrations than normal-weight individuals [57], and this could depend on the establishment of a condition of leptin resistance. Accordingly, a significant positive correlation was found in obese subjects between leptin levels and body weight, body fat percentage, body mass index (BMI), and insulin resistance [58,59]. This suggests that under obesogenic conditions, hyperleptinemia is associated with leptin resistance, which further contributes to the development of obesity and associated metabolic disorders. However, no additional increase was observed in obese patients with type 2 diabetes compared to non-diabetic obese subjects [60].

On the other hand, adiponectin is known to directly promote glucose metabolism and insulin sensitivity [61,62], increasing the oxidation of FAs, reducing the TG content in skeletal muscle and liver, and suppressing the hepatic production of glucose [63]. Adiponectin also exerts anti-inflammatory and anti-apoptotic effects and mitigates oxidative stress in different cell types [64,65,66]. Obese subjects show significantly lower levels of adiponectin than normal and overweight subjects [29]. It is worth noting that insulin-sensitive obese patients have significantly higher adiponectin levels than insulin-resistant patients with a similar BMI and waist circumference [67], suggesting the existence of a strong correlation between adiponectin levels and insulin sensitivity [68]. Interestingly, plasma levels of adiponectin appear to be progressively reduced in obese patients, obese patients with impaired fasting glycemia (IFG)/mild diabetes, and obese patients with T2D [41,68,69]. Recently, adiponectin has been proposed as a predictive marker of T2D in obese individuals even years before the onset of the disease [70].

Other adipokines whose levels are higher in obese subjects compared to normal-weight individuals include resistin [71,72], visfatin [73,74], apelin [30,75,76], the fatty acid binding protein specific for adipocytes (FABP4) [77,78], adipsin [79,80], and irisin [81,82].

Although the role of resistin in obesity and insulin resistance in humans is still highly debated [83], it has been found that resistin levels are higher in obese subjects than control subjects and significantly correlated with high adiposity and low insulin sensitivity [84]. In a recent meta-analysis, it was found that resistin levels in obese subjects with T2D were positively correlated with insulin resistance in subjects with hyperresistinemia but not in patients with normal circulating resistin levels [31]. Only one study has demonstrated that resistin levels are higher in obese women with diabetes than obese women without diabetes [85]. Further investigations are needed to determine the role of this adipokine in obesity and diabetes.

Visfatin plasma concentrations are significantly higher in obese subjects than normal-weight subjects and are reduced in obese subjects after weight loss [73]. On the other hand, the implication of visfatin in disorders of glucose homeostasis is still controversial. Indeed, although several studies show that visfatin levels are higher in obese patients with T2D compared to non-diabetic obese patients [86,87,88,89], they do not differ between obese subjects with newly diagnosed glucose metabolism disorders (impaired fasting glucose, impaired glucose tolerance, or T2D) and obese subjects without these abnormalities [90]. In addition, not all studies have found a positive correlation between visfatin levels and insulin resistance or metabolic syndrome [91,92,93]. However, since it has been demonstrated that visfatin levels are increased both in T2D patients [94,95,96] and by hyperglycemia or insulin resistance induced by FFA infusion, whereas they are reduced by hyperinsulinemia [97,98], the increase in visfatin levels under diabetic condition may be the result of insulin deficiency (as occurs in long-lasting β-cell dysfunction) or its inability to suppress visfatin production in insulin-resistant conditions [97,99]. Indeed, in patients with longer-standing T2D and endogenous insulin deficiency, visfatin concentration is increased with progression of β-cell dysfunction and worsening of glycaemia control [99]. Concordantly, Dogru et al. [100] found higher visfatin levels in diabetic patients than controls, without differences between non-obese patients with newly diagnosed diabetes and newly diagnosed impaired glucose tolerance (IGT), as well as between patients with IGT and healthy controls.

Apelin levels are increased in obese subjects [30,75,76] as well as in subjects without severe obesity but with IGT or overt diabetes [101]. In addition, levels of apelin are higher in obese subjects without diabetes than in obese patients with T2D [102]. In high-fat diet (HFD)-fed mice, apelin administration exerts beneficial effects on glucose metabolism and insulin sensitivity, and enhances the browning of WAT in both human and murine adipocytes [103,104]. Apelin levels probably increase during obesity to compensate for a state of insulin resistance, hyperinsulinemia, and impaired glucose metabolism. In addition, it is known that insulin can upregulate apelin expression in both human and murine adipocytes [30], suggesting the existence of a regulating loop where hyperinsulinaemia may promote apelin secretion during obesity as a compensatory mechanism to adapt to the enhanced insulin request.

FABP4, also known as adipocyte protein 2 (AP2), is a cytoplasmic fatty acid chaperone expressed mainly in adipocytes and macrophages. Although initially thought to be only a cytoplasmic protein, FABP4 was discovered in the bloodstream, and its levels were found to be higher in pathological conditions such as obesity, diabetes, and metabolic syndrome [77,105]. FABP4 plays a crucial role in the regulation of glucose/lipid metabolism and insulin sensitivity: *FABP4*-null mice are protected from HFD-induced hyperglycaemia, hyperinsulinaemia, and insulin resistance [106,107]. In addition, FABP4 contributes to the inflammatory response induced by macrophages in AT in the context of obesity [108]. In humans, a loss of function mutation in the *FABP4* gene determines a significantly reduced risk of developing T2D [109]. In obese subjects, circulating levels of this protein are significantly increased and positively correlated with adiposity indicators (BMI and percentage of fat), insulin resistance index (HOMA-IR), fasting glucose, and insulin levels [77,78]. In addition, obese subjects with newly diagnosed T2D show higher FABP4 levels compared to obese non-diabetic patients [110].

Adipsin/complement factor D, a member of the serine protease family, controls the alternative complement pathway and was one of the first adipokines described in 3T3 adipocytes [111]. Adipsin levels were found to be increased in obese patients compared to non-obese subjects [79,80], while they were reduced in patients with T2D compared to non-diabetic controls [112,113]. In addition, no changes in adipsin levels were found in obese subjects without diabetes compared to obese patients with T2D [70].

Irisin is a myokine, first described by Boström et al. [114] in 2012, which is mainly secreted by skeletal muscle in response to physical activity and a HFD. Capable of encouraging energy expenditure, irisin promotes the browning of WAT [115]. It is worth noting that irisin can be considered as an adipokine since it can also be secreted by WAT [116,117]. Most studies reveal that irisin levels are higher in obese subjects [81,82], and positively correlate with markers of adiposity [118,119], reflecting a condition of irisin resistance or a compensatory increase for metabolic abnormalities and insulin resistance. Concordantly, we have previously demonstrated that acute stimulation with saturated fatty acids (SFAs) increased irisin release from myotubes, and that mice receiving a HFD and gaining weight displayed an early increase in serum irisin levels [120]. On the other hand, most clinical studies, including meta-analyses, agree that circulating irisin levels are lower in patients with T2D, obese or not [82,121,122,123,124], probably reflecting a loss of the compensatory response following greater metabolic impairment. Interestingly, exogenous administration of recombinant irisin in animal models of diabetes and/or obesity improves glucose and lipid metabolism, thus showing antidiabetic and antiobesity effects [125,126,127].

It has been shown that angiotensinogen (AGT) can be secreted by AT, and its secretion is increased in both obese humans and obese mice [128]. Accordingly, obesity is also characterised by high levels of AGT and angiotensin-converting enzyme (ACE) [129]—two fundamental components of the renin-angiotensin system (RAS)—which plays a central role in the regulation of blood pressure and energy homeostasis. RAS is a complex system through which AGT is converted to angiotensin I by renin and then to angiotensin II (AngII) by ACE. AngII can be further transformed into angiotensin 1-7 (Ang1-7) by ACE2. AngII and Ang1-7 show antagonistic activity: AngII is proinflammatory, profibrotic, and has vasoconstrictive effects; whereas Ang1-7 exhibits anti-inflammatory, antifibrotic, and vasodilator effects, improving the lipid profile and insulin resistance [130,131]. Circulating levels of AngII were also found to be higher in obese hypertensive subjects and obese hypertensive subjects with T2D, suggesting that AngII represents a risk factor for hypertension associated with obesity [132]. On the other hand, it has been shown—particularly in animal models—that Ang1-7 counter-regulates the effects of AngII, and that activation of the Ang1-7/ACE2 arm leads to an improvement in the context of obesity and related diseases [131,133,134]. Interestingly, in obese children, Ang1-7 is inversely correlated with weight, BMI, and blood pressure; therefore, this metabolite has been proposed as a new biomarker in childhood obesity [135].

Dipeptidyl peptidase-4 (DPP-4) is a serine protease known for its ability to inactivate numerous hormones, including the incretin hormones glucagon-like peptide-1 (GLP-1) and glucose-dependent insulinotropic polypeptide (GIP) [136]. DPP-4 inhibitors are an available class of anti-diabetes agents, widely used for the treatment of T2D [137]. DPP-4 exists both bound to the cell membrane and in a systemically soluble form [138]. In 2011, DPP-4 was proposed as a new adipokine involved in the link between adipose tissue, obesity, and metabolic syndrome [139]. The release of DPP-4 is strongly correlated to the size of adipocytes, with an expression 5 times higher in VAT than SAT in obese subjects [139]. Serum DPP-4 levels are significantly higher in obese than normal-weight subjects, and are positively associated with fasting blood glucose and insulin concentrations as well as with insulin resistance index (HOMA-IR) [140,141,142]. In addition, no significant differences in DPP-4 levels were found between obese patients with T2D and obese non-diabetic subjects [142], although the difference in the enzymatic activity rather than in circulating levels should be evaluated. Indeed, the increase in plasma DPP-4 levels observed in obese patients with type 2 diabetes compared to non-obese diabetic subjects was not accompanied by differences in DPP-4 activity. This suggests that AT-derived DPP-4 contributes to increasing DPP-4 plasma concentrations but might not significantly add to the active pool of plasma DPP-4 [143].

Finally, in obese subjects, in addition to the altered pattern of adipokines secretion, AT releases greater quantities of FFAs [144], which are associated with insulin resistance and lipotoxicity [144,145]. In particular, while some studies report an increase in the levels of SFAs (i.e., palmitic acid [146], decanoic acid, and caprylic acid [147]), in obese subjects, unsaturated fatty acids levels have been found to be both decreased (i.e., oleic acid [147] and linoleic acid [146,148]) and increased (i.e., palmitoleic acid [146,149], dihomo-gamma-linolenic acid [149], and docosaesaenoic acid [147]).

Overall, AT secretes a high number of adipokines. The serum levels of many of these adipokines are altered in obese patients compared to normal-weight controls and sometimes in obese patients with T2D compared to non-diabetic obese subjects (Table 1). Importantly, it is difficult to determine which AT depot (SAT or VAT) mostly contributes to changes in serum adipokine levels during obesity, since increased/decreased adipokine mRNA or protein levels in adipocytes do not always reflect their increased/decreased serum concentrations.

Interestingly, many of these adipokines might directly influence numerous aspects of β-cell function and viability, including insulin synthesis and secretion, as well as β-cell apoptosis and proliferation [14,15,16]. Therefore, it is to be expected that alterations in the levels of these adipokines could contribute to the reduction in the β-cell functional mass, which is chiefly responsible for the onset of T2D.

## 3. Dysfunctional Adipose Tissue Secretome Affects β-Cell Functional Mass: The Natural History of an Announced Failure

The dysregulation of the β-cell functional mass, which is the reduction in the number of β-cells and their ability to secure adequate insulin secretion, represents a key mechanistic factor linked to the onset and progression of T2D [151]. During obesity, the nutrient surfeit leads to the development of hyperglycaemia and hyperlipidaemia, which increases the metabolic load and promotes the ectopic storage of lipids in different tissues—including the pancreas [152]—triggering insulin resistance and chronic inflammation [153]. Under these conditions, β-cells are initially characterised by a marked plasticity: when insulin sensitivity in peripheral tissues is reduced, insulin secretion is increased to ensure euglycemia [154]. As a consequence, while obesity and insulin resistance remain major risk factors for T2D, the compensatory capacity of β-cells is credited in preventing most obese and insulin-resistant subjects from developing T2D [155]. Indeed, several studies have demonstrated that the β-cell mass is increased by approximately 20–90% in overweight/obese non-diabetic subjects compared to lean controls [156,157,158]. Correspondingly, obese non-diabetic subjects show elevated fasting plasma insulin levels and a several-fold greater insulin response to glucose stimulation, both in vivo and ex vivo (reviewed in [155,159]).

In mice, this compensatory response is sustained by the enhancement of insulin synthesis and secretion as well as by the stimulation of β-cell proliferation, which increases the β-cell mass [159]. However, β-cells in humans have a limited ability to proliferate. Thus, the compensatory expansion of β-cell functional mass in response to insulin resistance could be attributable to other mechanisms, such as β-cell neogenesis from duct cells, increased β-cell size, and trans-differentiation from α- to β-cells [155,160,161]. Over time, if uncorrected, the prolonged and concurrent exposure to high glucose and FFA levels (glucolipotoxicity) exhausts the adaptive capacities of β-cells, promoting β-cell dysfunction and ultimately their loss. Under these conditions, insulin secretion is no longer able to compensate for the reduced insulin sensitivity, and IGT and overt T2D are sequentially established [159]. Indeed, in T2D patients, the β-cell function might be reduced already by 50% at diagnosis [162], while β-cell mass is reduced by 25–65% compared to non-diabetic subjects [155,159].

**Figure 1:**
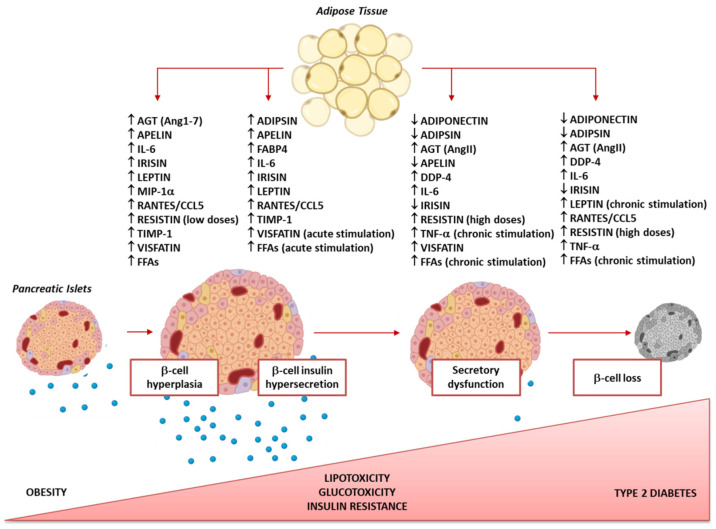
Timeline of the possible alterations in the adipokines secretion pattern in the transition from obesity to T2D that reflects the progressive deterioration of β-cell functional mass. ↑, increased levels; ↓, decreased levels. AGT, angiotensinogen; AngII, angiotensinogen II; DPP-4, dipeptidyl peptidase-4; FABP4, fatty acid-binding protein specific for adipocytes; FFAs, free fatty acids; IL-6, interleukin-6; MIP-1α, macrophage inflammatory protein-1α; RANTES, regulated upon activation normal T cells, expressed and secreted; TIMP-1, tissue inhibitor of metalloproteinases-1; TNF-α, tumour necrosis factor-α.

Overall, the natural history of β-cell failure in obesity-induced T2D can be divided into three steps: (1) β-cell compensatory hyperplasia and insulin hypersecretion, (2) insulin secretory dysfunction, and (3) loss of β-cell mass. In the current review, we summarise the evidence about the ability of the AT secretome to influence each step of β-cell failure and attempt to draw a timeline of the alterations in the adipokine secretion pattern in the transition from obesity to T2D that reflects the progressive deterioration of the β-cell functional mass (Figure 1).

### 3.1. How Adipose Tissue Secretome Influences β-Cell Compensatory Hyperplasia

The β-cell mass is finely regulated by at least five independent mechanisms [163]: (1) β-cell proliferation, (2) β-cell size changes, (3) β-cell neogenesis, (4) β-cell apoptosis, and (5) β-cell identity (i.e., β-cell dedifferentiation [164,165] and β- to α-cell trans-differentiation [160,166]). Therefore, β-cell hyperplasia may be due to increased β-cell proliferation (especially in mice), size, or neogenesis from ductal cells, as well as to α- to β-cell trans-differentiation. The mechanisms by which obesity may drive β-cell compensatory hyperplasia are still nearly unknown. Numerous studies have suggested the ability of several adipokines to regulate β-cell proliferation.

Among these potential adipokines, leptin has been demonstrated to induce proliferation of the mouse pancreatic β-cell line MIN6 [167], rat insulin-secreting β-cell line RINm5F [168], and foetal rat pancreatic islets [169] by signalling via the leptin receptor [168,169] and probably through the activation of a mitogen-activated protein kinase (MAPK) [167]. Surprisingly, Morioka et al. showed that mice knockout for the leptin receptor in the pancreas exhibited a significantly reduced islet mass (61.8% of control) after an HFD [15]. Similarly, the pancreatic islets of Zucker diabetic fatty (ZDF) rats—which are leptin receptor-deficient and spontaneously develop T2D—are normal in the pre-diabetic state and characterised by disorganised architecture and increased β-cell death (~50% lower β-cell mass) after diabetes occurs [170,171]. Furthermore, leptin-resistant diabetic mice accumulate large quantities of triacylglycerol in β-cells, even on a diet containing only 6% fat. Under these conditions, β-cells appear to be disorganised and interspersed with abundant fibrous tissue, and the β-cell mass is reduced [172]. In that leptin has been reported to regulate lipid metabolism in β-cells and protect them from the effects of lipid overload [173], leptin-resistant mice exhibit greater β-cell mass loss when receiving an HFD, possibly because they are characterised by a more pronounced lipotoxicity in β-cells [15]. In fact, the islets from ZDF rats with diabetes have more than 50 times the amount of triglycerides than pre-diabetic ZDF rats [174]. However, it should be recognized that, due to the complex metabolic condition of the ZDF rat, it is difficult to draw firm conclusions about the role of the leptin receptor specifically in β-cells, based on the studies conducted in this animal model.

Similarly, resistin increases cell viability in mouse βTC-6 and rat BRIN-BD11 β-cell lines, but only at physiological concentrations of 10–20 ng/mL. At higher pathological concentrations associated with obesity and diabetes (30–40 ng/mL), this increase in cell viability was not seen [175]. These results suggest that resistin may contribute to β-cell hyperplasia, but only at low concentrations.

Irisin promotes β-cell proliferation in INS-1E cells, probably through activation of the extracellular signal-regulated kinase (ERK)1/2 and p38 MAPK pathways [120,176], and improves the β-cell mass and proliferation in healthy wild-type mice [115,120]. Visfatin [177] and apelin [178] are also able to increase β-cell proliferation in the mouse pancreatic β-cell line MIN6 [177] and HFD-streptozotocin (STZ)-induced diabetic rats [178], respectively. MIP-1α and RANTES/CCL5 have the potential to promote β-cell proliferation, although to date this has only been demonstrated when they are secreted, along with other cytokines/chemokines, by T cells that infiltrate pancreatic islets in type 1 diabetes or insulitis [179]. Their role in obesity-induced T2D has not been proven. Finally, TIMP-1 enhances the replication of pancreatic islets β-cells and preserves β-cell mass in animal models of diabetes [180,181].

Overall, this evidence suggests that several adipokines, differentially expressed during obesity, can promote β-cell proliferation. However, as already discussed, β-cells in humans have a limited ability to proliferate. Therefore, the compensatory expansion of the β-cell mass could be attributable to other mechanisms, such as β-cell neogenesis from duct cells, increased β-cell size, and trans-differentiation from α- to β-cells [155,160,161]. Unfortunately, to the best of our knowledge, only a few studies have explored the possibility that some adipokines may influence these processes.

Among the relevant adipokines, it has been shown that apelin can effectively reduce β- to α-cell trans-differentiation and maintain β-cell identity in STZ-induced diabetic and HFD-fed mice [182], ensuring the maintenance of the β-cell mass. These islet effects were coupled with decreased β-cell apoptosis in both models, and there was an accompanying increase in β-cell proliferation in STZ-induced diabetic mice [182]. Accordingly, mice knockout for the apelin receptor in the pancreas showed reduced islet size and density as well as reduced β-cell mass [183]. In addition, the obesity-induced adaptive elevations in mean islet size and fractional islet area were significantly reduced in knockout mice when compared with wild-type mice [183]. Similar results on pancreatic β-cell mass have been obtained in the HFD-fed STZ-induced experimental type 2 diabetic rats receiving a once-daily intraperitoneal injection of apelin (0.1 μmol/kg) for 10 weeks [184]. Together, these findings demonstrate an important role for apelin in the regulation of pancreatic β-cell hyperplasia.

Transgenic mice overexpressing IL-6 show islet hyperplasia and an increased number of extra- and intra-islet ducts, suggestive of islet neogenesis [185]. It is probable that these effects are mediated by the IL-6-induced expression of the islet neogenesis associated protein (INGAP) in pancreatic acinar tissue [186]. Some other studies have revealed a regenerative property of IL-6 [187], thus suggesting that IL-6 elevation observed in obesity may be one of the underlying mechanisms through which β-cells self-adapt and regenerate under stressful inflammatory conditions [188]. Since IL-6 has always been considered a pro-inflammatory cytokine, the discovery that IL-6 exerts beneficial effects on β-cells may seem somewhat paradoxical. However, it is possible that IL-6 may act in a bimodal way according to its concentration and biological context [189].

Although the increase in AGT levels during obesity would not seem to have a direct effect on β-cell mass, its metabolite Ang1-7 exerts beneficial effects on β-cell survival and identity. It has been demonstrated that Ang1-7 improves β-cell survival in rodent models of diabetes (HFD-fed mice and HFD-fed/STZ-injected rats) [190,191], protects β-cell lines from palmitate-induced apoptosis [192], and attenuates pancreatic β-cell dedifferentiation in an HFD-fed mouse model [193].

In addition, several adipokines have been shown to reduce β-cell apoptosis induced by various harmful stimuli (e.g., FABP4 [194], leptin [195,196], irisin [120,176,197], visfatin [177,198], apelin [182], TIMP-1 [180,181,199,200], IL-6 [201,202,203,204], adipsin [205]). However, reduced apoptosis cannot properly be considered as a mechanism by which compensatory hyperplasia of β-cells occurs; rather, it could be a mechanism that prevents β-cell loss.

Significantly, acute exposure to FFAs also stimulates β-cell proliferation [206] and may promote compensatory β-cell hyperplasia [207] regardless of the nature of the FFAs. Indeed, treatment with FFAs increases β-cell proliferation in rat islets [208,209], and intralipid infusion into normal rats increases β-cell mass and proliferation [210,211]. Therefore, at an early stage of obesity, an increase in FFAs might drive a compensatory increase in β-cell mass. However, whether in vivo exposure to lipids promotes β-cell proliferation remains questionable, and how this relates to human biology is unclear [212].

### 3.2. How the Adipose Tissue Secretome Influences β-Cell Compensatory Insulin Hypersecretion

During obesity, an increase in plasma insulin concentrations occurs both under basal conditions and postprandially. Concordantly, non-diabetic obese subjects show elevated fasting plasma insulin levels and a several-fold greater insulin response to glucose stimulation both in vivo and ex vivo [155,159]. Insulin hypersecretion is generally considered as an initial adaptive response to compensate for the reduced insulin sensitivity that often characterises obesity [154,155,159], and it is sustained by the enhancement of both insulin synthesis and secretion in mice [155]. It is noteworthy that recent work demonstrated that the increase in insulin secretion in obese subjects occurs even in the absence of insulin resistance, thus representing an early abnormality that precedes and contributes to the development of insulin resistance [213].

Although the mechanisms by which obesity may drive β-cell compensatory insulin hypersecretion are still nearly unknown, numerous studies have suggested the ability of the AT secretome to regulate the β-cell insulin secretory function.

Leptin might play a role in β-cell compensatory insulin hypersecretion, particularly in the state of leptin resistance that occurs in obesity. Indeed, although the leptin effect on the insulin secretory function could be influenced by the experimental model, concentration, timing, and environmental conditions (such as glucose concentration in the medium; reviewed in [214]), several studies to date have shown that leptin has a potent direct inhibitory effect on insulin biosynthesis and secretion, both in vitro and in vivo [215,216,217,218,219,220,221,222,223,224,225,226]. On the other hand, the synthesis and release of leptin are physiologically stimulated by insulin, and this gives rise to a hormonal regulatory loop called the ‘adipo-insular axis’ [227,228]. During obesity, the state of leptin resistance and the increase in leptin levels could contribute to dysregulation of the adipo-insular axis, including at the level of the pancreatic β-cell, thus promoting the development of hyperinsulinemia and disturbances of glucose metabolism in overweight patients [215,226].

The increase in IL-6 levels during obesity has been linked to β-cell compensatory insulin hypersecretion as it can act directly on pancreatic β-cells, enhancing glucose-stimulated insulin secretion (GSIS) without affecting insulin content [229]. Accordingly, it has been demonstrated that IL-6 can increase insulin secretion in HIT-T 15 cells [230] and rat pancreatic islets with no effects on the insulin content of the islets [231]. It is worth noting that IL-6 administration or elevated IL-6 concentrations in response to exercise can also stimulate GLP-1 secretion from intestinal L cells and pancreatic α-cells, thus indirectly improving insulin secretion and reducing glycemia. In models of T2D, the beneficial effects of IL-6 were maintained, while IL-6 neutralisation resulted in a further elevation of glycemia and reduced pancreatic GLP-1 [232]. This finding suggests that IL-6 released by AT can mediate the compensatory insulin hypersecretion under conditions of obesity both directly, by stimulating insulin secretion, and indirectly, by increasing islet GLP-1 production [232].

Similar to IL-6, the increase in irisin levels during obesity could mediate insulin secretion adaptation in response to increased insulin demand. Indeed, among its pleiotropic effects, irisin increases proinsulin mRNA levels, insulin content, and GSIS in human and murine islets, in rat and human β-cell lines, and in vivo when intraperitoneally administered to healthy mice [120]. Accordingly, serum irisin levels were closely related to β-cell function (measured using homeostasis model assessment index-β, HOMA-β) in normal glucose tolerance subjects, suggesting that irisin may play a crucial role in pancreatic β-cell function [233]. Notably, irisin was also able to prevent β-cell dysfunction under glucotoxic and lipotoxic conditions [120,197]. All these data support the hypothesis that irisin may be a compensatory mechanism to offset HFD/obesity-induced insulin resistance by increasing energy expenditure [234,235] and insulin secretion to prevent diabetes.

A role in coordinating the β-cell response to obesity has also been suggested for FABP4, whose levels are raised during obesity. On the one hand, FABP4 can potentiate GSIS in isolated mouse islets and mice in vivo without affecting insulin content, although this activity does not acutely occur and manifests over a longer period. Accordingly, circulating FABP4 levels correlated with GSIS during obesity in humans. On the other hand, insulin inhibited FABP4 release from adipocytes in vitro as well as in mice and humans, consistent with a feedback regulation [236]. Although these data suggest the existence of an endocrine circuit between FABP4 and insulin, which could coordinate the response of β-cells to obesity, there is a lack of further studies confirming the role of this endocrine loop in β-cell compensatory insulin hypersecretion during obesity. It could be possible that FABP4 levels increase in obese patients because insulin is no longer able to inhibit FABP4 release due to the insulin resistance that occurs in obesity.

RANTES/CCL5 is an interesting chemokine whose levels are increased during obesity is. Although it is implicated in the pathogenesis of diabetes by promoting immune cell recruitment through activation of C-C chemokine receptors (CCRs), RANTES/CCL5 also shows beneficial effects on β-cells through the activation of the G-protein-coupled receptor 75 (GPR75) [237]. Accordingly, RANTES/CCL5, through the activation of the CCRs, reduces glucose-stimulated GLP-1 secretion in the human enteroendocrine cell line NCI-H716 and in mice in vivo, resulting in impaired insulin secretion after glucose stimulation in mice [238]. On the other hand, via GPR75, RANTES/CCL5 stimulates insulin secretion from β-cell lines [239] and mouse and human pancreatic islets in vitro, and improves glucose tolerance in lean mice and a mouse model of hyperglycaemia and insulin resistance without changing insulin sensitivity [237]. Interestingly, it has been demonstrated that adiponectin can negatively regulate the expression of RANTES/CCL5 [240]. As a consequence, the reduction in adiponectin levels during obesity results in increased RANTES/CCL5 levels, which should induce an increase in insulin secretion.

Other adipokines with an insulinotropic effect and whose levels increase in obese patients include adipsin and TIMP-1. Adipsin knockout mice show glucose intolerance due to insulinopenia, and their islets are characterized by reduced GSIS ex vivo [241]. Of interest, adipsin levels are reduced in T2D patients with β-cell failure, while exogenous administration of adipsin to diabetic mice reduced hyperglycemia by boosting insulin secretion [205,241]. Interestingly, the complement component C3a, whose generation is induced by adipsin, exerts a potent insulin secretagogue effects, and the C3a receptor is required for insulinotropic effects of adipsin [241]. Regarding TIMP-1, it has been demonstrated that coculture of murine islets—stimulated with STZ to reduce secretory function—with human umbilical cord mesenchymal stem cells overexpressing TIMP-1 increased insulin and C-peptide secretion [242]. Moreover, stimulation with TIMP-1 prevented cytokine-mediated inhibition of GSIS in rat islets [199]. Despite this finding, it has been demonstrated that TIMP-1 deficiency does not affect GSIS in mice in vivo [243].

Visfatin’s role on insulin secretion remains controversial. Although several studies have demonstrated that this adipokine (or the product of its reaction, the nicotinamide mononucleotide, NMN) has an insulinotropic action and a protective role on β-cell function [244,245,246,247], a recent study showed that visfatin can act in a bimodal and dose-dependent way. Indeed, at low physiological levels, visfatin maintains β-cell function and identity; however, as its levels rise, as in T2D, it induces β-cell dysfunction and apoptosis, as well as reduced β-cell identity [248]. Similarly, in human islets, both visfatin and NMN potentiated GSIS only when administered for an acute period, with no effects for a longer time of stimulation [249]. Although there are no data regarding the effects of increased visfatin levels on β-cells during obesity, we could speculate that it may exert initial beneficial effects that may be lost when visfatin levels become chronically increased.

A bimodal and dose-dependent action has been demonstrated also for apelin, which inhibits GSIS at low doses and has no effects [250] or increases GSIS at elevated doses [251]. As stated above (Section 2), insulin enhances apelin expression in human and mouse adipocytes [30], suggesting the existence of a regulating loop that may promote apelin secretion during obesity as a compensatory mechanism to adapt to enhanced insulin requests. The role of apelin remains controversial [252,253], in part due to its rapid plasma degradation. Therefore, experiments are carried out with apelin analogues, which could show different pharmacodynamic characteristics compared to the endogenous molecule [254].

A positive role on β-cell function has been proposed in recently published papers for the AGT-derived metabolite Ang1-7 in both prediabetic (HFD-fed mice and rats) and diabetic (STZ-injected rats) animal models, and β-cell lines [192,255,256,257,258,259].

Finally, a controversial role is also played by FFAs. Although FFAs acutely stimulate insulin release in pancreatic β-cells [260,261], chronic elevation results in impaired insulin secretion [262]. Importantly, the effects of FFAs on β-cell function depend on their chemical nature, dose, time of exposure, interaction with other nutrients [263,264], as well as their binding to the G protein-coupled receptors (GPCRs) expressed on β-cell surface (i.e. GPR40, 41, 43, and 120) [265]. Since different GPCRs show specificities for FFAs of differing chain length and degree of saturation, they can differently modulate the effects of FFAs on β-cell function. For instance, despite the existence of conflicting results, several studies demonstrated that GPR40 and GPR120, which are typically activated by medium and long-chain FAs (both saturated and unsaturated), can potentiate insulin secretion [265]. It is generally accepted that unsaturated fatty acids have a lower dysfunctional role in β-cells compared to SFAs [264,266,267]. In addition, it has been widely demonstrated that combining SFAs with unsaturated fatty acids confers protection from SFAs-induced dysfunction [268,269,270,271,272].

### 3.3. How the Adipose Tissue Secretome Influences β-Cell Insulin Secretory Dysfunction

β-cell insulin secretory dysfunction, defined as the loss of the ability of pancreatic β-cells to produce and release insulin in concentrations sufficient to maintain euglycemia, occurs when the high and prolonged secretion of insulin in response to prolonged environmental insults leads to the exhaustion of pancreatic β-cells [154,155,159].

Several studies suggest that the pathogenesis of β-cell secretory dysfunction can be strongly influenced by several adipokines. Among these adipokines, the reduction in the levels of adiponectin that occurs during obesity strongly contributes to β-cell dysfunction. Indeed, it has been demonstrated that adiponectin can enhance insulin secretion by promoting exocytosis of insulin granules, and upregulating the expression of the *Insulin* gene, although this effect depends on the glucose concentration and extent of insulin resistance [16,273,274,275]. Accordingly, adiponectin had no significant effect on insulin secretion and potentiated GSIS in islets from lean mice, yet it reduced basal insulin secretion in islets from obese mice [276]. Similarly, in INS1 cells, adiponectin had a mild inhibitory effect on forskolin-enhanced GSIS, whilst it partially rescued defective insulin secretion in cytokine- and FFA-treated cells [277]. In addition, adiponectin can stimulate insulin secretion at low physiological glucose concentrations in mouse islets in vitro, and the injection of adiponectin stimulates insulin secretion in vivo [273]. Although it has been reported that adiponectin does not affect insulin secretion in human islets at either basal or stimulatory glucose concentrations [278], and that there is no association between circulating insulin and adiponectin levels in humans [279], it has also been demonstrated that low adiponectin levels are associated with β-cell dysfunction [280]. Under this last concept, adiponectin levels in humans are significantly and positively associated with the disposition index. In particular, a positive association between adiponectin levels and insulin secretion was identified with an index incorporating an adjustment for insulin resistance [281]. In sum, these data suggest that the reduction in adiponectin could mediate β-cell dysfunction during obesity.

Furthermore, the increase in IL-6 levels observed during obesity can result in a 75% decrease in adiponectin secretion, as shown in 3T3-L1 adipocytes [282]. IL-6 also downregulates adiponectin mRNA expression in a reversible, time- and dose-dependent manner [282]. This suggests that although IL-6 could play a direct role in β-cell compensatory insulin hypersecretion (Section 3.2), it could also indirectly contribute to β-cell secretory dysfunction.

TNF-α has shown an inhibitory effect on the insulin secretory capacity of pancreatic β-cells. Specifically, chronic (but not acute) stimulation of rat INS-1E cells with TNF-α decreases GSIS without changing the total amount of insulin [283]. Accordingly, TNF-α seems not to influence insulin gene expression in human β-cell cultures in vitro [284]. In HIT-T15 cells, TNF-α suppressed both basal and glucose-stimulated insulin transcription and secretion, and this effect was significantly enhanced by high glucose levels [285]. Notably, it has been demonstrated that TNF-α increased the expression of adhesion molecules on β-cells, and reversibly perturbed the typical segregation between β-cells and non-β-cells within the pancreatic islets, thus altering islet architecture and influencing insulin secretion [286]. However, although obese subjects have higher average circulating levels of TNF-α in the plasma than lean controls, these levels are considered well below those required to reduce insulin secretion [12]. Therefore, according to some Authors, a direct endocrine role for TNF-α appears to be somewhat unlikely in β-cell secretory dysfunction during obesity. On the other hand, it has been demonstrated that TNF-α can stimulate a profound increase in IL-6 production in 3T3-L1 differentiated adipocytes [287], hence its indirect role on β-cellular dysfunction could be assumed.

The increase in circulating levels of DPP-4 occurring during obesity could determine a pronounced inactivation of endogenous GLP-1, thus reducing the insulinotropic action of GLP-1 and consequently insulin secretion. In addition, DPP-4 could directly reduce insulin secretion regardless of GLP-1 [288]. As a consequence, several studies have shown that the inhibition of DPP-4 increases insulin secretion in various experimental models [289,290,291,292], including human β-cells of T2D patients [288].

Several studies have investigated the role of resistin in the regulation of β-cell function. Adenoviral-mediated overexpression of resistin in mice caused defective GSIS, and treatment of rodent β-cells and isolated islets with resistin impaired GSIS in a dose-dependent manner [293,294,295]. Furthermore, resistin impaired insulin signalling in mouse β-cells and islets [175,293], thus impairing β-cell mass and function [296,297]. In human islets, however, the data remain controversial [298,299]. The existence of a bimodal effect of resistin has been theorised that it could exert a positive role on β-cell function at lower physiological concentrations as part of an adipo-insular axis to maintain functional β-cell mass under an adipotoxic challenge, with a negative role at higher concentrations [175]. Notably, the resistin-to-adiponectin ratio (RA index)—instead of resistin alone—emerged as strongly associated with β-cell function, since in vitro experiments and data on humans in vivo revealed a negative correlation between the RA index and insulin secretion. Interestingly, unlike the RA index, adiponectin levels alone were not associated with insulin secretion [300].

With some exceptions [301,302,303,304], several studies have shown the detrimental role of AGT-derived AngII on insulin release in vitro, ex vivo, and in vivo, both in animals and humans, which is at least partially mediated by reductions in proinsulin biosynthesis [305,306,307,308,309,310,311,312]. Concordantly, the inhibition of the RAS system has been demonstrated to be responsible for increased insulin biosynthesis and release in several obese, prediabetic, or diabetic models [313,314,315,316,317,318,319,320,321].

Interestingly, for some adipokines, involvement in both β-cell compensatory insulin hypersecretion and insulin secretory dysfunction could be presumed according to their circulating levels. For example, we have already discussed that the increase in irisin levels during obesity could play a role in β-cell compensatory insulin hypersecretion (Section 3.2). On the other hand, the reduction in irisin levels observed in patients with long-lasting obesity and T2D [82,121,122,123,124] could play a causal role in insulin secretory dysfunction.

As discussed above (Section 3.2), visfatin acts in a bimodal and dose-dependent way [248,249]; thus, the increase in its levels observed in long-lasting obesity and T2D induces a diabetic phenotype in pancreatic islets [50].

Similarly, apelin is able to both increase GSIS at elevated doses (Section 3.2) or inhibit it at low doses [250,251,252,253]. Therefore, as insulin can stimulate apelin secretion [30], it could be hypothesised that when insulin levels begin to drop, apelin levels are not more stimulated and also show a decrease [30], contributing to insulin secretory dysfunction.

Chronic high levels of circulating FFAs, particularly SFAs, could represent one of the main causes of β-cell dysfunction [263,264]. SFAs levels, in particular palmitic acid, are elevated in obese patients, and correlate with a risk of developing T2D [322]. Notably, chronic elevated palmitic acid levels have detrimental effects on β-cell function by reducing both GSIS and the insulinotropic effects of the incretin hormones [323,324,325]. A detrimental role has also been demonstrated for saturated stearic acid [326].

Finally, the involvement of extracellular vesicles (EVs) in the cross-talk between AT and pancreatic β-cells has recently been explored. Indeed, EVs from healthy 3T3-L1 adipocytes can promote insulin secretion in INS-1E cells and human pancreatic islets under basal conditions or exposed to cytokines/glucolipotoxicity, whereas EVs from inflamed adipocytes caused β-cell dysfunction. Human lean adipocyte-derived EVs produced similar beneficial effects, whereas obese adipocyte-derived EVs were harmful to human EndoC-βH3 β-cells [327].

### 3.4. How the Adipose Tissue Secretome Influences Loss of β-Cell Mass (Apoptosis/Dedifferentiation)

Since β-cell apoptosis was shown to be increased in patients with T2D without changes in β-cell replication or neogenesis, β-cell loss should be the main mechanism responsible for the reduced β-cell mass in T2D subjects [156]. Nevertheless, more recent studies have suggested that β-cell dedifferentiation, as well as trans-differentiation from β- to α-cells, may contribute to β-cell loss in T2D [164]. Numerous studies have suggested the ability of several adipokines to promote the loss of β-cell mass, particularly through the induction of apoptosis. Interestingly, the adipokines that promote β-cell loss are often the same ones that improve β-cell compensatory hyperplasia (e.g., leptin, IL-6, resistin, RANTES/CCL5; Section 3.1).

For example, it has been demonstrated that chronic exposure of human islets to leptin decreases β-cell production of the anti-apoptotic interleukin-1 (IL-1) receptor antagonist while inducing the release of the pro-apoptotic cytokine IL-1β from the islet preparation, thus leading to caspase-3 activation and β-cell apoptosis [328]. Similarly, Maedler et al. demonstrated that chronically elevated concentrations of leptin and glucose-induced β-cell apoptosis through the activation of the c-jun N-terminal kinase (JNK) pathway in human islets and rat insulinoma (INS 832/13) cells [329]. In addition, leptin was able to induce inflammation-related genes in RINm5F insulinoma cells [330]. On the other hand, it is known that an increase in leptin levels due to a condition of leptin resistance often occurs in obese patients [331]; therefore, the inability of leptin to exert its proliferative and anti-apoptotic effects at the β-cellular level could also be responsible for β-cell loss under these conditions.

Similar to leptin, IL-6 can induce β-cell apoptosis [332,333] and—together with TNF-α and the non-adipokine IL-1β—promote β-cell dedifferentiation in cultured human and mouse islets [334]. These findings suggest that IL-6 can also contribute to β-cell mass loss. Likewise, as stated above (Section 3.3), resistin may exert a bimodal effect at β-cellular levels, and be responsible for both β-cell hyperplasia and β-cell loss according to its concentration [175]. It has been demonstrated that a high dose of resistin induces rat insulinoma cell RINm5F apoptosis [335] and downregulates insulin receptor expression levels (necessary for the maintenance of β-cell mass) in clonal β-cells, hence decreasing cell viability [175]. RANTES/CCL5 equally exerted a marked pro-apoptotic action in clonal β-cells [336] and murine pancreatic islets [337]. The bimodal action of these cytokines could depend on the dynamics of their secretion, concentration, and exposure time [189,338]; therefore, their increased levels in obesity can exert both initially beneficial and then deleterious effects on pancreatic β-cells.

Furthermore, while the AGT-derived Ang1-7 exerts a beneficial effect on β-cell survival and regulation of the β-cell mass (as described in Section 3.1), the AGT-derived metabolite AngII promotes β-cell apoptosis. Indeed, AngII blockers have been shown to promote β-cell regeneration and improve β-cell mass recovery in rodent models of diabetes [318,321,339,340,341,342,343]. Notably, the RAS system may also promote β-cell dedifferentiation [344], thus contributing to β-cell mass loss.

As previously mentioned (Section 3.1; [120,176,197]), irisin exerts anti-apoptotic and proliferative effects in rodent and human β-cell lines, as well as in mouse and human pancreatic islets, both in vitro and in vivo. However, irisin levels are reduced in T2D compared to non-diabetic controls [82,121,122,123,124], and this reduction may contribute to β-cell loss. Similarly, adiponectin has been shown to increase β-cell proliferation [218,274,345] and reduce β-cell apoptosis [65,218,277,345] in rodent β-cell lines and in mice models. Consistent with these results, overexpression of full-length adiponectin maintains β-cell mass and glucose homeostasis in *ob/ob* mice and a model of type 1 diabetes [65,346,347]. Unfortunately, adiponectin levels are reduced in obese and T2D patients [29,41,67,68,69], thus promoting β-cell loss. Accordingly, adiponectin null mice show a significant reduction in β-cell proliferation rates and β-cell areas [348] and are more susceptible to β-cell apoptosis [65].

Similarly to irisin, adipsin is able to reduce β-cell death (Section 3.1 [205]), and therefore the reduction in its levels that occurs in T2D patients [112,113] may contribute to β-cell loss. Indeed, it has been demonstrated that chronic replenishment of adipsin in diabetic *db/db* mice preserves β-cell mass by blocking β-cell death and dedifferentiation, thus increasing insulin levels and ameliorating hyperglycemia [205].

In addition to β-cell dedifferentiation [334], TNF-α has also been shown to induce apoptosis in rodent and human pancreatic β-cells [349,350,351] and various β-cells lines [352,353,354,355,356,357]. Furthermore, as mentioned (Section 3.3), TNF-α increases the expression of adhesion molecules on β-cells and perturbs the typical segregation between β- and non-β-cells, thus altering islet architecture and influencing cell survival [286]. Nevertheless, as already mentioned for the β-cell function (Section 3.3), Zhao et al. asserted that an elevation in TNF-α in obese humans and animals does not reach levels concomitant with those known to deleteriously affect β-cell survival [12].

DPP-4 is also expected to indirectly reduce β-cell proliferation and survival by inactivating GLP-1 and then reducing its beneficial effects at the β-cellular level [338]. Indeed, the use of DPP-4 inhibitors exerts both direct and indirect beneficial effects on β-cell mass [151]. In particular, it has been shown that the DPP-4 inhibitor linagliptin restores β-cell turnover in human islets and the human β-cell EndoC-βH1 exposed to stressful stimuli [288,290]. Similarly, the inhibition of DPP-4 restored islet cell mass in a rodent model of T2D [292,358]. These data suggest that the increase in DPP-4 during obesity could significantly contribute to β-cell loss.

Finally, chronic elevation in the circulating concentration of FFAs represents one of the main causes of β-cell mass loss (reviewed in [322,359]). Importantly, as stated for β-cell function (Section 3.3), FFAs’ quality—not only quantity—might also impact β-cell function [322]. For example, saturated palmitic acid is more toxic than monounsaturated oleic acid and polyunsaturated linoleic acid in rodent and human β-cells [322,360], while combining palmitic acid with oleic acid confers protection from palmitic acid-induced apoptosis [361,362].

## 4. Future Perspectives and Concluding Remarks

The cross-talk between AT and pancreatic β-cells could represent the missing link connecting obesity to T2D. Growing evidence describes AT as an endocrine organ that produces a large number of adipokines that can influence the function of multiple tissues [11,12,13], including numerous aspects of β-cell function and viability [14,15,16]. The dysregulation of the β-cell functional mass represents a key mechanistic factor linked to the onset and progression of T2D [151]. The natural history of β-cell failure in obesity-induced T2D can be divided into three steps: (1) β-cell compensatory hyperplasia and insulin hypersecretion, (2) insulin secretory dysfunction, and (3) loss of β-cell mass. In the current review, we have summarised the evidence about the ability of AT secretome to influence each of these steps and have attempted to draw a timeline of the alterations in adipokine secretion pattern in the transition from obesity to T2D that reflects the progressive deterioration of β-cell functional mass (Figure 1). Thereby, the adipokine secretion pattern could both become a new early biomarker of β-cell suffering and help discriminate between obese patients at risk of developing diabetes from those not at risk, thus suggesting when prompt drug intervention is needed.

Unfortunately, the topic remains extremely complex and not definitively resolved: many adipokines act in a bimodal manner, and can exert both beneficial and detrimental effects on β-cell function and survival depending on their concentration, time of exposure, and surrounding environment (such as the case of apelin [250,251], IL-6 [189], resistin [175], visfatin [248,249], and FFAs [206,207,260,261,262,322,359]). In addition, it could be possible that several adipokines could influence each other or show additive or neutralising effects. Therefore, it might be erroneous to study the effects of a single adipokine without considering the influence of the others.

In addition, it is important to underline the existence of a gender-dependent dimorphism in the adipokine levels secreted by adipose tissue (e.g. higher levels of adiponectin, leptin, and visfatin in women than in men) [363]. These variations could be due to both differences in the distribution of adipose tissue (more visceral and hepatic AT in men, more subcutaneous AT in women) and influences of sex hormones [363,364,365,366]. Whether this gender dimorphism may affect changes in adipokine levels during obesity and/or diabetes and then influence β-cell functional mass is possible but not yet known.

Finally, the study of the AT secretome and its alteration during obesity could help in better understanding the mechanisms linking obesity to β-cell dysfunction and death, finally leading to T2D, and to identify new promising molecules (e.g., adipokines) to halt the loss of β-cell functional mass.

## Figures and Tables

**Table 1:** Changes in adipokine levels in obese patients compared to normal-weight controls, and in obese patients with type 2 diabetes compared to non-diabetic obese patients. Adipokines are listed in alphabetical order.

Adipokines	Obese vs. Normal-Weight	Obese with T2D vs. Non-Diabetic Obese	Refs.
Adiponectin	↓	↓	[29,41,68,69]
Adipsin	↑	=	[70,79,80]
AGT (AngII, Ang1-7)	↑ (↑, ↓)	N/A	[128,129,132,135]
Apelin	↑	↓	[30,75,76,101,102]
DPP-4	↑	=	[140,141,142]
FABP4	↑	↑	[77,78,110]
GRO-alpha	↑	N/A	[28,47]
IL-6	↑	↑	[35,37,41]
Irisin	↑	↓	[81,82,121,122,123,124]
Leptin	↑	=	[57,60]
MCP-1	↑	N/A	[36,40]
MIP-1α and MIP-1β	↑	N/A	[27,50,51,52]
PAI-1	↑	N/A	[49]
RANTES/CCL5	↑	N/A	[45]
Resistin	↑	↑	[71,72,84,85]
TIMP-1	↑	N/A	[28,48]
TNF-α	↑	↑	[35,38,42,150]
TPO	↑	N/A	[28]
Visfatin	↑	↑	[73,74,86,87,88,89]

↑, increased levels; ↓, decreased levels; =, no changes; N/A, not available. AGT, angiotensinogen; AngII, angiotensinogen II; DPP-4, dipeptidyl peptidase-4; FABP4, fatty acid-binding protein specific for adipocytes; GRO-alpha, growth-related oncogene factor-alpha; IL-6, interleukin-6; MCP-1, monocyte chemoattractant protein-1; MIP-1β, macrophage inflammatory protein-1β; PAI-1, plasminogen activator inhibitor-1; RANTES, regulated upon activation, normal T cells expressed and secreted; T2D, type 2 diabetes; TIMP-1, tissue inhibitor of metalloproteinases-1; TNF-α, tumour necrosis factor-α; TPO, thrombopoietin.

## Data Availability

Not applicable.
